# Barriers to obtaining prostate multi‐parametric magnetic resonance imaging in African‐American men on active surveillance for prostate cancer

**DOI:** 10.1002/cam4.2149

**Published:** 2019-05-21

**Authors:** Eric L. Walton, Mustafa Deebajah, Jacob Keeley, Shadi Fakhouri, Grace Yaguchi, Milan Pantelic, Craig Rogers, Hakmin Park, Mani Menon, James O. Peabody, Ali Dabaja, Shaheen Alanee

**Affiliations:** ^1^ Wayne State University School of Medicine Detroit Michigan; ^2^ Vattikuti Urology Institute Henry Ford Hospital Detroit Michigan; ^3^ Department of Radiology Henry Ford Hospital Detroit Michigan

**Keywords:** health services accessibility, magnetic resonance imaging, prostate cancer

## Abstract

**Purpose:**

Magnetic resonance imaging is playing an ever‐bigger role in the management of prostate cancer. This study investigated barriers to obtaining multi‐parametric MRI (mpMRI) in African‐American men on active surveillance for prostate cancer in comparison to white men affected by the same type of cancer.

**Materials and Methods:**

Retrospective review of prostate mpMRI orders from August 2015 to October 2017 at a single health organization treating a diverse population was performed. Data was extracted from the electronic medical records and cancellations were examined based on the documented reason for mpMRI cancellation, race, median zip code household income, and distance from healthcare facility.

**Results:**

Out of 793 prostate mpMRI orders, 201 (25%) went unscanned. Access to care issues accounted for 46% of unscanned orders. Patient cancellations were the most common, followed by difficulty contacting patients, and insurance denials. African‐American patients disproportionately went unscanned because institution staff were unable to contact patients (29% vs 10% in white men, *P* = 0.0015). Median zip code household income was significantly different between racial groups but did not vary between indication for cancellation.

**Conclusions:**

African‐American prostate cancer patients' access to mpMRI is hindered more by barriers to care than White patients. Urology providers must consider these issues before using prostate mpMRI within their active surveillance pathways.

## 1 INTRODUCTION

Among men, prostate cancer is the most common nondermatologic cancer and one of the top three causes of cancer death.^1^ Transrectal ultrasound‐guided (TRUS) biopsy is the current standard diagnostic procedure for prostate cancer; this method randomly samples the prostate with a sensitivity as low as 60%.^2^ In recent years, the utilization of multiparametric magnetic resonance imaging (mpMRI) has paved the way for MRI‐targeted biopsy (TB). With this approach, patients with highly suspicious lesions on mpMRI are counseled on the benefit of MRI‐TB and given the option of undergoing a biopsy of the prostate. In the published literature, compared with TRUS biopsy, mpMRI has higher sensitivity and negative predictive value for clinically significant cancer (Gleason score 4 + 3 or higher, volume ≥0.5 cm, or aggressively invasive cancers).^3^ As such, mpMRI identifies significant cancers—those that require treatment—and protects up to 25% of patients from the risks of an unnecessary biopsy.^3^, ^4^

The American Urological Association (AUA) recognizes the usefulness of mpMRI in pre‐biopsy risk stratification of patients being evaluated for prostate cancer. It correlates well with the likelihood of clinically significant lesions and likelihood of progression on active surveillance.^5^ In a large, prospective active surveillance cohort, unchanged mpMRI had an 80% negative predictive value for biopsy upgrade to clinically significant cancer.^6^ Lack of standardization in repeat mpMRI for surveillance is a limitation in its use for in monitoring men on active surveillance. Therefore, while the AUA considers mpMRI beneficial for stratifying men into active surveillance protocols, they recommend a combination of molecular markers, repeat mpMRI imaging, and biopsy for monitoring men on active surveillance.^5^

Considering growing evidence supporting the advantages of MRI‐TB over the ultrasound‐guided approach, it is important to understand barriers limiting patients from pursuing this diagnostic approach. This study aimed to identify reasons and barriers for incomplete unscanned prostate mpMRIs in patients who are scheduled to undergo MRI‐TB at a large community health organization. Accessing a large African‐American population has enabled our organization to examine race as a determining factor in a patient's chance to receive mpMRI during the course of active surveillance for prostate cancer.

## 2 MATERIALS AND METHODS

This is a retrospective review of prostate mpMRI ordered from August 2015 to October 2017 at a large health care organization treating a diverse population the metro Detroit area. The urology department at this institution manages 1800 patients with prostate cancer on a yearly basis, 35% of whom identify as African American. After institutional review board approval, data were extracted from the electronic medical records (EMRs) with case by case review by researchers to confirm the accuracy of the data. All prostate mpMRI orders during the study dates were identified and extracted into a database.

Variables collected included date the mpMRI was ordered, date of birth, ethnicity, zip code, indication for mpMRI, and documented reason for mpMRI cancellation. Patients were excluded from analysis if they did not have complete information. Patient age was calculated from date of birth and date the mpMRI was ordered. Median household income was extracted by zip code from the 2016 American Community Survey. Distance from hospital was calculated as the distance between the center of the patient's zip code and the healthcare institution where patients were seen by providers. Unscanned mpMRIs were categorized based on the documented reason for mpMRI cancellation, the primary outcome of interest in this study. Categories were “patient cancellation,” “provider cancellation,” “not contacted,” “scanned at outside institution,” “medical condition,” and “insurance.”

Descriptive statistics for categorical variables included frequencies and proportions. Chi‐square tests with Pearson residuals were utilized for statistical analysis of categorical variables. Analysis of variance (ANOVA) with Tukey's honest significance tests (HSD) were utilized for statistical analysis of continuous variable. Univariate and multivariate logistic regression using African American and White patients within 100 miles of the hospital was also performed with SAS 9.4 in order to identify variables significantly associated with completed mpMRI. Tests were two‐sided with an alpha value of 0.05.

## 3 RESULTS

During the study period ranging from August 2015 to October 2017, 793 prostate mpMRIs were ordered. Of these, 749 had complete information extracted from the EMR. A total of 201 25%) mpMRI orders were never completed, 175 with complete information available in the EMR. Patient characteristics for scanned and unscanned orders are displayed in Table 1; patients with incomplete orders ranged in age from 37 to 88 years old, with 68% identifying as White and 28% identifying as African American. Unscanned orders were categorized based on indication for mpMRI cancellation. Table 2 displays frequencies by ethnicity for each indication.

**Table 1:** Characteristics of patients with an EMR order placed for prostate mpMRI

Characteristic	Scanned (n = 574)	Unscanned (n = 175)	*P*‐value
Age—mean (SD)	65.4 (8.9)	65.4 (8.4)	1.000
Ethnicity, n (%)			
White	388 (67.6)	119 (68.0)	0.5003^a^
African American	150 (26.1)	49 (28.0)	
Other	36 (6.27)	7 (4.0)	
Median zip code household income in thousands of USD—mean (SD)	64.8 (25.4)	65.6 (25.8)^b^	0.7089
White	70.9 (25.6)	71.9 (24.3)	0.7002
African American	47.9 (23.9)	49.9 (28.1)	0.6395
Other	75.1 (25.6)	69.6 (31.7)	0.6183

a Χ^2^ (2, N = 749) = 1.38.

b ANOVA: F = 12.7861, *P* < 0.0001.

**Table 2:** Indications for cancelled prostate mpMRI by racial group

Ethnicity	Indication—n (% of the column)					
	Provider cancelled	Patient cancelled	Medical condition	Not contacted	Went off‐site	Insurance
White	37 (88%)	23 (83%)	16 (57%)	11 (44%)	12 (67%)	15 (68%)
African American	5 (12%)	5 (17%)	12 (43%)	14 (56%)	6 (33%)	7 (32%)

Χ^2^ (5, N = 165) = 19.58, *P* = 0.0015.

Among African‐American patients whose mpMRIs went unscanned, the most common cause of not undergoing imaging was difficulty contacting patients. Difficulty contacting patients, insurance denials, and patient cancellations are all barriers to care. Combined, this makes access to care issues (46%) the most common indication for an unscanned prostate mpMRI. Among these three barriers patient cancellation was most common overall, followed by difficulty contacting patients and insurance denials. Other reasons for cancellation included medical contraindications (16%) and imaging offsite (13%).

Indication for cancellation was not equally distributed between racial groups [Χ^2^ (5, N = 165) = 19.58, *P* = 0.0015]. Pearson residuals indicate prostate mpMRIs of African‐American patients disproportionately went unscanned because it was difficult to contact patients (29% vs 10% in white males); prostate mpMRIs of White patients were disproportionately cancelled by providers. Among patients unable to be contacted, a median of two attempts were made without a statistical difference between racial groups (F = 0.8988, *P* = 0.551). Provider cancellations occurred 56% of the time due to duplicate or incorrect EMR order. Therapy was escalated (ie, radiation or directly to biopsy) in five cases and de‐escalated in two cases due to decreased prostate‐specific antigen (PSA).

Using median household incomes from subjects' zip codes, the average household income of the unscanned study population was $65 558, which did not differ from the scanned population that had an average household income of $64 808 *P* = 0.7002). Median zip code household incomes stratified by ethnicity are displayed in Table 1 and by indication for cancellation in Table 3. African‐American patients live in zip codes with a median household income statistically lower than White patients and patients of unknown ethnicity *F* = 12.7861, *P* < 0.0001). On average, the median zip code household income of African‐American patients was $49 853 but the median zip code household income of White patients was $71 892 and the median household income of other ethnicities was $69 625. However, within each ethnic group the median household income did not differ between the scanned and unscanned populations. Patients who went off‐site were from wealthier zip codes $73 393) and patients who were not contacted or have medical contraindications to mpMRI live in less wealthy areas $62 824 and $61 803, respectively). Table 4 demonstrates this pattern is consistent among African American patients, but not White patients. That being said, the median zip code household income was not statistically different from indication for cancellation, even when separated by ethnicity.

**Table 3:** Median zip code household income in USD by indication for cancellation

Indication for cancellation	Median Zip code household income in USD—mean (SD)
Patient cancelled	68 568 (21 077)
Not contacted	62 824 (33 876)
Went off‐site	73 393 (24 539)
Medical condition	61 803 (31 230)
Insurance	66 748 (33 693)
Provider cancelled	68 030 (22 620)

ANOVA: F = 0.6780, *P* = 0.6406.

**Table 4:** Median zip code household income in USD by indication for cancellation amongst African American and White patients

African American patients—mean (SD)		White patients—mean (SD)	
Patient cancelled	54 818 (23 107)	Patient cancelled	68 889 (19 879)
Not contacted	47 213 (33 740)	Not contacted	74 421 (21 119)
Went off‐site	60 499 (25 279)	Went off‐site	73 605 (25 219)
Medical condition	40 538 (16 932)	Medical condition	73 252 (28 365)
Insurance	51 854 (39 631)	Insurance	78 698 (31 332)
Provider cancelled	59 064 (25 968)	Provider cancelled	69 811 (22 538)
ANOVA: F = 0.5816, *P* = 0.7138		ANOVA: F = 0.4050, *P* = 0.8444	

Racial groups were distributed similarly between the scanned and unscanned populations. This remained true when nonaccess to care explanations ie, went offsite, duplicate order, incorrect order) for unscanned mpMRI were excluded from analysis. Univariate logistic regression, displayed in Table 5, did not identify any association between patient age, ethnicity, median zip code household income, or distance from the healthcare institution and scanned mpMRI order. This was confirmed with multivariate logistic regression for each of these variables, displayed in Table 6.

**Table 5:** Univariate logistic regression analysis of 612 patients for completion of mpMRI scanning after order placement SAS 9.4

Predictor	β	SE β	Wald's χ^2^	*df*	*P*‐value	Odds ratio
Intercept	1.5062	0.8243	3.3389	1	0.0677	4.509
Age	0.0014	0.0125	0.0120	1	0.9128	1.001
Intercept	1.5447	0.1137	184.73	1	<0.0001	4.687
African American ethnicity	−0.1517	0.1137	1.7826	1	0.1818	0.859
Intercept	1.3893	0.2749	25.534	1	<0.0001	4.012
Household income	0.0000	0.0000	0.6385	1	0.4243	1.000
Intercept	1.2376	0.2107	34.496	1	<0.0001	3.447
Distance from care	0.0173	0.0090	3.7284	1	0.0535	1.017

**Table 6:** Multivariate logistic regression analysis of 603 patients for completion of mpMRI scanning after order placement SAS 9.4

Predictor	β	SE β	Wald's χ^2^	*df*	*P*‐value	Odds ratio
Intercept	1.0498	0.8908	1.3887	1	0.2386	2.857
Age	0.0033	0.0127	0.0660	1	0.7972	1.003
African American ethnicity	−0.0831	0.1339	0.3850	1	0.5349	0.920
Household income	−0.0000	0.0000	0.0265	1	0.8707	1.000
Distance from care	0.0164	0.0102	2.5586	1	0.1097	1.017

## 4 DISCUSSION

Access to care has been defined by five dimensions: availability, accessibility, accommodation, affordability, and acceptability.^7^ Some of the important barriers to access to care include difficulty contacting patients which represent a failure in the realm of accommodation, or the manner in which a medical facility accepts patients and the ability of clients to meet those constraints. Another barrier is insurance denials which represent a failure within affordability. Patient cancellations likely occurred due to failures in any of the five dimensions. That is, it is reasonable to expect patient cancellations because they cannot find an acceptable appointment time (availability); do not have transportation (accessibility); cannot navigate the healthcare system (accommodation); cannot afford the procedure (affordability); or they dislike the facility (acceptability). In this study, we are the first to report that barriers to access to care were the most common indication for unscanned prostate mpMRI orders, and that these barriers affected African‐American patients more than White patients treated in the same metro area.

In this study, African‐American patients struggled with access to care more than White patients. This is reflected by the disproportionate number of African‐American patients with prostate mpMRIs cancelled due to difficulty contacting the patient. PSA screening among African‐American patients supports the idea that access to care issues contribute to this finding. African‐American patients who found the healthcare system inconvenient or who found it difficult to receive quality care were less likely to complete a PSA test.^8^

It has been shown that patients' socioeconomic status (SES) is often the primary cause for radiological study cancellation.^9^ For example, SES is a major barrier for patients to obtain screening tests for breast cancer; patient awareness and knowledge about breast cancer has been reported as a barrier to obtaining a mammography in older women regardless of financial status.^10^, ^11^ Evidence points to health disparities in all fields of medicine, including urology. Individuals of lower SES travel farther for care, receive care less often at high‐volume institutions, and present with higher grade prostate cancer.^12^, ^13^, ^14^

From a SES perspective, epidemiological evidence indicates African‐American residents of Metropolitan Detroit disproportionately inhabit lower SES neighborhoods. Statistically lower median household income in the zip codes of the African‐American patients in this study—compared with White patients—supports this idea. 15, 16 Furthermore, lower SES individuals engage with digital health technology less than their higher SES counterparts.^17^ Therefore, it is reasonable to extrapolate that decreased engagement with health technology could explain communication difficulties amongst African‐American patients. Interestingly, in our study, median zip code household income was not significantly different when patients were grouped by indication for cancellation or when compared between African‐American patients with complete and incomplete mpMRI orders. This suggests that while lower income may play a role in why African‐American patients were successfully contacted less frequently than their White counterparts, it does not explain all variation. Another explanation for the lack of association between lower income and the decreased likelihood of mpMRI scanning is the small sample size of our study population precluding enough power to detect significant difference in the variables of interest.

Regarding implicit bias, substantial evidence suggests physicians and lay persons have similar degrees of bias. Both groups have higher levels of bias towards non‐White individuals, which can unconsciously have a negative impact on treatment adherence, patient‐physician relationships, and health outcomes.^18^ Thus, it is feasible that implicit bias may influence which patients' healthcare staff struggled to contact. It is unlikely healthcare staff invested less energy to contact African‐American patients than White patients as they made the same average number of attempts at communication. Furthermore, the staff responsible for patient communication at the study institution are overwhelming African American and female, two demographics known to show lower levels of implicit bias. 19 It's also possible African American patients unconsciously felt distanced from their providers and elected to not participate in further communication. Future investigation of this health disparity might benefit from a measure of implicit bias, however, inclusion of that type of measure in this study was not possible due to its retrospective nature.

Provider cancellations were the most common individual reason for an unscanned prostate mpMRI among White patients. This categorization includes duplicate orders, incorrect orders (eg, liver and prostate mpMRI changed to prostate mpMRI), and clinical judgement (eg, replaced with surgery/TRUS biopsy). Duplicate and incorrect orders account for over half of cancellations in this category. This is not an unexpected finding as cancellations due to duplicate orders tend to increase with EMR implementation.^20^ As such, provider cancellation likely reflects either user error, unfamiliarity with the EMR system, or increased radiologist recognition of duplicate orders. Importantly, they do not reflect a barrier to care because they are logistical errors that do not influence patient outcomes.

Among the remaining indications for prostate mpMRI cancellation, only insurance denials represent a social issue. This signifies a need within the urological community to produce further research and advocacy campaigns that compel insurance companies to cover MRI‐TB. Medical contraindications primarily encompass obesity, claustrophobia, and implants incompatible with the MRI machine. The former two can be resolved with open MRI machines and anxiolytics, respectively. Finally, offsite MRI is not truly a barrier to care as patients can still proceed with MRI‐TB once medical records are shared between institutions.

Overall, we think this study provides important insight into the difficulty of active surveillance pathways that utilize mpMRI as a way to manage African‐American patients, which is ensuring compliance with follow‐up. In fact, our patients who could not be accessed to attend their mpMRI were not compliant with the rest of their visits for active surveillance (data not shown). Based on the results of this study and pending validation of our findings from other similar healthcare systems, urology providers may want to strongly consider addressing access to care barriers when ordering prostate mpMRI for their patients, specifically those patients who identify as African American. Providers may also want to consider investigating whether patients feel their healthcare facility is available, accessible, accommodating, affordable, and acceptable. Resolving issues in these five dimensions will reduce the number of patient cancelled prostate mpMRIs, which are the most common barrier to imaging and the most common reason for cancellation (if duplicate orders are excluded). Providers need to develop methods to ensure their clinic staff can contact all patients prior to schedule appoints, especially African American patients who are disproportionately affected by this barrier. Part of this may require accommodations to meet the needs of lower SES patients. In addition, insurance barriers should be addressed for all patients, as this represents a small, yet clinically significant segment of unscanned prostate mpMRIs. Finally, active surveillance pathways that are specifically designed for patients who may not be able to overcome barriers to care are in dire need, and active treatment may be a better option if the provider objective assessment of the patient leads them to believe that safe active surveillance management is not achievable in that particular patient.

A limitation of this study is that we could not account for the intrinsic bias in deciding who an MRI should be ordered for based on our retrospective methods. In patients who obviously would not have been able to attend the MRI (for financial reasons, transportation reasons, etc), the provider may have decided to just not order the mpMR, and the patient would not be captured in this study. Another limitation because of our retrospective design is if patients had previously had an MRI ordered at another institution and did not therefore need an MRI ordered at our institution, then these patients would also not be captured in the group of patients who obtained access to an MRI. To try to correct for this limitation, we reviewed the patients' records through CareEverywhere in EPIC©, an electronic medical record that allows us to retrieve patients past medical history from the major medical systems in Michigan, and very few record showed evidence of previous MRI orders. These few orders of MRI outside our system would not have affected the results of our analysis, and in fact may bias our conclusions towards showing African Americans face more barriers than White patients, which was a conclusion of the study. Also, not undergoing mpMRI could be related to lack of understanding of the value of the test, mistrust, or lack of education by the provider which are factors that we did not measure in this study. Finally, there are multiple reports regarding the racial disparities in the United States regarding screening, care and outcomes in prostate cancer, but we feel that the growing role of MRI in managing prostate cancer calls for an analysis of the quality of access of African‐American men to this important diagnostic tool.

## 5 CONCLUSIONS

When obtaining mpMRI in patients considering active surveillance for prostate cancer; African‐American patients are more impacted with barriers to access to care than White patients. As a result, urology providers must consider access to care issues in African‐American patients before recommending prostate mpMRI.

## CONFLICT OF INTEREST

The authors have no conflicts of interest to disclose.

## AUTHOR CONTRIBUTIONS

Eric L Walton: Data curation, writing—original draft. Mustafa Deebajah: Data curation, writing—original draft. Jacob Keeley: Data curation, formal analysis, methodology, writing—review and editing. Shadi Fakhouri: Data curation. Grace Yaguchi: Data curation. Milan Pantelic: Data curation, writing—review and editing. Craig Rogers: Supervision, writing—review and editing. Hakmin Park: Data curation, writing—review and editing. Mani Menon: Supervision, writing—review and editing. James Peabody: Supervision, writing—review and editing. Ali Dabaja: Data curation, methodology, project administration, resources, supervision, writing—original draft. Shaheen Alanee: Data curation, methodology, project administration, resources, supervision, writing—original draft.
